# Computing stoichiometric molecular composition from crystal structures

**DOI:** 10.1107/S1600576714025904

**Published:** 2015-01-30

**Authors:** Saulius Gražulis, Andrius Merkys, Antanas Vaitkus, Mykolas Okulič-Kazarinas

**Affiliations:** a Vilnius University Institute of Biotechnology, Graiciuno 8, LT-02241 Vilnius, Lithuania; b Vilnius University Faculty of Mathematics and Informatics, Naugarduko 24, LT-03225 Vilnius, Lithuania

**Keywords:** molecular structure, multimolecular ensembles

## Abstract

Crystallographic investigations deliver high-accuracy information about positions of atoms in crystal unit cells. For chemists, however, the structure of a molecule is most often of interest. The structure must thus be reconstructed from crystallographic files using symmetry information and chemical properties of atoms. Most existing algorithms faithfully reconstruct separate molecules but not the overall stoichiometry of the complex present in a crystal. Here, an algorithm that can reconstruct stoichiometrically correct multimolecular ensembles is described. This algorithm uses only the crystal symmetry information for determining molecule numbers and their stoichiometric ratios. The algorithm can be used by chemists and crystallographers as a standalone implementation for investigating above-molecular ensembles or as a function implemented in graphical crystal analysis software. The greatest envisaged benefit of the algorithm, however, is for the users of large crystallographic and chemical databases, since it will permit database maintainers to generate stoichiometrically correct chemical representations of crystal structures automatically and to match them against chemical databases, enabling multidisciplinary searches across multiple databases.

## Abstract 2

An algorithm to compute stoichiometrically correct molecular formulae from crystal structures is proposed. The algorithm’s output is suitable for high-volume automated searches in chemical databases and for linking crystallographic and chemical information.

## 1. Introduction   

In many applications, there is a need to compute a molecular structure and composition from a crystal structure solved by diffraction methods. Crystallographic structure files describe the structure of the asymmetric unit and provide symmetry operators to restore the unit cell and the whole crystal structure from the minimal unique set of atoms. Since a molecule of high symmetry can often be located on a special position, such molecules are represented only by a part of their atoms. To obtain the full set of atoms for such a molecule, symmetry-equivalent atoms must be generated by using the crystal symmetry operators and tracing the networks of covalently connected atoms. Such algorithms are implemented in free open-source programs like *Jmol* (Hanson, 2010 ▶) or proprietary ones like *enCIFer* (Allen *et al.*, 2004 ▶). Other programs, like *Avogadro* (Hanwell *et al.*, 2012 ▶) or *Open Babel* (O’Boyle, Banck *et al.*, 2011 ▶), rely on pre-generated molecular structures, although in principle there is nothing that would prevent adding symmetry-handling algorithms as plugins or as main code libraries.

**Figure 1:**
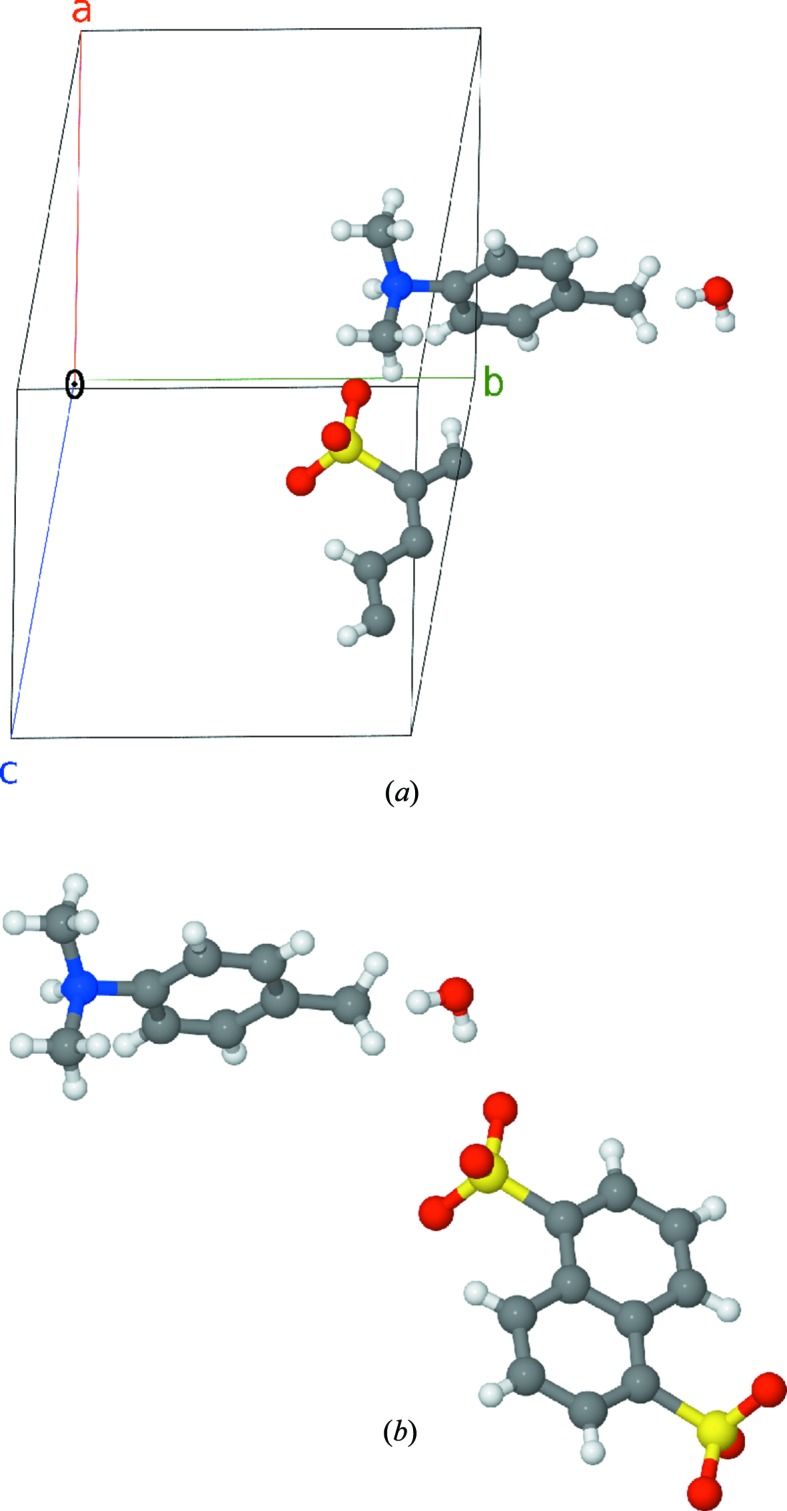
Reconstructions of molecular moieties using the simple algorithm. (*a*) The asymmetric unit of the crystal provided by the COD 2231955 (Wei, 2011 ▶) entry. (*b*) Molecules reconstructed using the *cif_molecule* implementation. Image generated using *Jmol* (Hanson, 2010 ▶) and *POV-Ray* (Persistence of Vision, 2004 ▶).

The existing algorithms, however, generate separate mol­ecules, disregarding their relative abundance in a crystal. We will refer to such an algorithm as a ‘simple reconstruction’ algorithm. To provide an example, our own implementation of this algorithm in the *cif_molecule* program from the *cod-tools* package (available at svn://www.crystallography.net/cod-tools/trunk under the GPL2 free software license) reconstructs one moiety for each distinct atom in the asymmetric unit of a crystal cell; all other programs that were inspected by us produce the same result. For example, from the entry 2231955 (Wei, 2011 ▶) from the Crystallography Open Database (COD; Gražulis *et al.*, 2009 ▶, 2012 ▶), one moiety of sulfonic acid and one moiety of amine in the default molecule reconstruction (Fig. 1 ▶) are produced.

While this is sufficient for on-screen viewing and visual analysis, a problem arises when the resulting molecular data are converted to files in chemical formats that no longer contain the unit cell and symmetry information. If chemical information such as molecular formula, charge balance, molecular weight or component molar ratios is computed from such files, the results will be wrong and thus will confuse users who do not know the origin of the file. For example, even a casual look at Fig. 1 ▶(*b*) reveals that a single positively charged amino group is accompanied by two negative charges of the sulfo groups. Unfortunately, such files would be occasionally generated by conventional algorithms if they were used in fully automated mode. It is therefore deemed desirable to employ an algorithm that produces output with correct stoichiometric ratios of all atoms, especially when atom coordinates are represented in chemical information files like SDF (Dalby *et al.*, 1992 ▶) that do not contain any crystallographic information.

## 2. Algorithm   

Clearly, all necessary information for the above-mentioned reconstruction of molecules is contained in a crystal structure [such as one expressed in a PDB (Berman *et al.*, 2012 ▶) file or CIF (Brown & McMahon, 2002 ▶)], since the correct summary chemical formula, representing the composition of the crystal formula unit at a given *Z* value (Hall *et al.*, 2005 ▶), can be readily obtained from atom multiplicities and occupancies. The multiplicities, if not given, can be computed from atomic coordinates and symmetry operators.

Thus, the only problem remaining is to determine which symmetry operators need to be applied to atoms so that (*a*) all complete molecules are reconstructed and (*b*) stoichiometric ratios between molecular components are preserved. To arrive at a suitable algorithm we observe that the problem with the COD 2231955 entry conversion arises because the inversion centre is applied to the naphthalene-1,5-disulfonate atoms, since this molecule is situated around a special position, but not to the dimethyl(4-methylphenyl)ammonium ion and a water molecule at general positions. If we applied the same inversion centre to the ammonium ion and the water molecule as we applied to the parts of the disulfonate, we would obtain the correct molecular composition.

There seem to be two approaches to solve this problem. The first one would be to expand the crystal asymmetric unit to the *P*1 unit cell and then remove excess molecules that are symmetry equivalent, keeping however the correct ratios of the molecules. The second approach would be to generate unique molecules, using symmetry-equivalent atoms if necessary, and then to apply those symmetry operators to the molecules that were not yet applied to them but were used to generate their partners in the crystal. The former approach is straightforward, but excessive molecules will be generated, only to be removed afterwards; the second approach requires some algebra but involves less computation and should therefore be faster. Both algorithms have been implemented, and a comparison will be given below.

In the first approach, all connected molecules in the unit cell are generated. Below we provide an informal ‘comments-type’ description of the algorithm; a formal working sample implementation in the Perl programming language (Wall *et al.*, 2000 ▶) can be found in the *cod-tools* package:

(1) All symmetry operators of the crystal space group are applied to each atom, and the image of each atom is reduced modulo 1, * i.e.* moved to a representative unit cell which is the unit cell closest to the origin in the first octant {this unit cell spans fractional coordinates 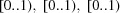 on the crystal axes}; each such image receives a unique ‘cell_label’ identifier, and a list of ‘cell_label’ identifiers used in molecules is set up, originally empty.

(2) Atoms in 26 adjacent unit cells are generated; to speed up neighbour search, all atoms from all 27 unit cells (the representative unit cell and the 26 cells adjacent to it) are distributed into an array of cubic ‘boxes’. Each box has a vertex equal to the longest possible covalent bond length, which in this case is twice the largest covalent radius of an atom in the unit cell plus some (configurable) safety margin [a strategy known at least since 1966 (Levinthal, 1966 ▶)]. Thus, to search for covalently bonded atoms only the 27 cubic boxes must be searched that are adjacent to the box containing the atom at which the bond is supposed to start. This is significantly less time consuming than searching all generated atoms in the 27 unit cells. The algorithm run time grows linearly with the number of atoms if atom density remains constant. It is implicitly assumed that the longest bond in the crystal is shorter than any side of a unit cell.

(3) An atom with a yet unused ‘cell_label’ is chosen as the starting point for the new molecule. All atoms connected to it are found in the ‘boxes’ and appended to the molecule atom list if they are not yet in this list. Then, for each new atom, this step is repeated recursively. When searching for neighbours of an atom that is not in the representative cell, the atom’s coordinates are reduced to this unit cell {*i.e.* fractional coordinates are reduced to the range 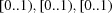 by taking their fractional parts}, and the neighbours are sought at the new position. The translation between the original and the reduced positions is afterwards added to the coordinates of the found neighbours. In this way, we make sure that we find also atoms that are outside the 27-unit-cell block. We stop constructing the molecule when there are no new atoms connected to it by covalent bonds (all neighbours of the newly added atom layer are either too far away or already in the molecule). We stop searching for new molecules when all unused atoms in the representative unit cell (as identified by ‘cell_label’ identifiers) are exhausted.

(4) In the molecule list generated by the previous step, symmetry-equivalent molecules generated by each symmetry operator are present with at least one of their atoms in the representative unit cell. Molecules fall in groups of symmetry-equivalent molecules. Each molecule in such a group is the symmetric image of the other molecule in the group. Not all of these images are needed for a minimal stoichiometrically correct description of the substance. Each molecular group can be identified as originating from the same atoms in the original CIF; therefore these atoms have the same site labels (from the 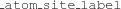 data item) in each equivalent molecule. We thus form molecular keys *K* by concatenating sorted site labels for each molecule and grouping together molecules with identical keys. We then count the number of molecules under each key and find a greatest common divisor *D* of the molecule counts. We output only 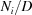 molecules from each group, where 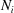 is the total count of molecules in the *i*th group, producing a stoichiometrically correct description of the substance.

(5) The stoichiometric description of a substance is not yet minimal, however, since a crystal may contain more than one chemically identical molecule in the asymmetric unit and all such (crystallographically non-equivalent) molecules will be present in the output. To reduce such duplication, a chemical fingerprint, for example a Morgan fingerprint (Morgan, 1965 ▶), can be used as a key for molecule grouping instead of the key *K* built from site labels in step 2. The Morgan algorithm introduces canonical numbering on the atomic graph. The fingerprint produced with its help would be (usually) different for covalently different molecules and equal for molecules with identical connectivities, and thus would allow one to recognize and remove chemically identical molecules, further simplifying the resulting molecular structure. Since, however, the chemical fingerprinting makes additional assumptions about molecular identity based on different chemical properties, the use of Morgan fingerprints has been made optional in the algorithm implementation.

The second approach is similar to the *P*1 algorithm described above but we do not use all atoms of the unit cell as molecule ‘seeds’; instead, in step 2 we only use atoms belonging to the asymmetric unit, as specified in the original CIF. We therefore find only a minimal set of molecules in which each molecule has at least one atom in the asymmetric unit. This set of molecules is not stoichiometrically correct, since some molecules may contain a symmetry-equivalent set of atoms while others will not. For example, application of this algorithm to the COD entry 2231955 will yield one naph­thal­ene-1,5-disulfonate and one dimethyl(4-methylphenyl)­ammonium moiety, like the other commonly used programs (Fig. 1 ▶). We observe that we have applied an inversion centre to the naphthalene disulfonate moiety atoms in order to generate the whole molecule from the asymmetric unit; therefore, to preserve stoichiometry, the same symmetry operator (in general, more than one operator) must be applied to the ammonium ion as well (and to all other molecules if such are present in the crystal), provided they were not used to generate these molecules.

To find the minimum set of symmetry operators to be applied additionally to each generated molecule, the symmetry group of each molecule must be determined. Afterwards, all symmetry groups will be multiplied, giving the symmetry group of the ‘molecular cluster’ in the crystal. To this end, an algorithm published by R. Grosse-Kunstleve for reconstruction of symmetry groups was used (Grosse-Kunstleve, 1999 ▶). To generate missing molecules of the molecular cluster, for each molecule we now find and apply operators that were not used for reconstructing this particular molecule; these operators do not belong to the symmetry group of the molecule and therefore must belong to a coset of the mol­ecule’s symmetry group in the cluster symmetry group. We must apply exactly one operator from each left coset of the mol­ecular symmetry group. We will show below that the molecular cluster generated in this way will have correct stoichiometric ratios of the molecules.

To begin, we demonstrate that we do not need to apply several operators from the same left coset. Indeed, let us assume that all atoms of a molecule *M* are mapped onto the symmetry-equivalent atoms of the same molecule by any molecule symmetry operators 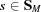, where 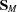 is the (point) symmetry group of the molecule, yielding the same physical molecule (with possibly permuted order of atoms): 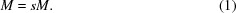
The molecular cluster symmetry group 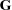 is formed by multiplying all possible combinations of symmetry operators of all individual molecule symmetry groups, building a supergroup. Let us now choose a symmetry operator 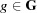 from the cluster symmetry group 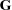 that does not belong to 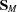. If such an operator exists, it does not map *M* to itself (otherwise it would be in 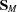); thus it maps molecule *M* to some other symmetry-equivalent image: 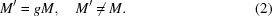



If we now choose another symmetry element 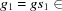 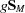 from the left coset 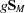, and apply it to *M*, we get 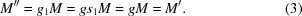



Thus, any operator 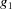 from the same left coset as *g* yields the same molecule image 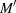.

Second, we demonstrate that application of one symmetry operator from each left coset yields a stoichiometrically correct molecular cluster. To show this we first note that the cluster symmetry point group 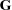 is a subgroup of the (finite) full group 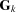 of the crystal under investigation and thus has an integer index 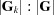 in this full group.^1^ The operators in 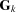 reconstruct one unit cell from the asymmetric unit, and since the translations of the crystallographic space group preserve stoichiometric relations within the unit cell, the application of 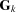 to the asymmetric unit must yield atoms in correct stoichiometric relations. Further, the application of the representative operators from the left coset of 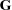 in 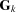 to the molecular cluster atoms yields disjoint molecular cluster images in distinct space points. Indeed, if some molecule in a cluster is mapped onto itself by an operator, then it belongs to a point symmetry group of that molecule. But, by the definition of 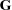, the group 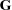 should contain this operator and should map the cluster to itself, because either it maps a cluster molecule to itself, or, by definition of the cluster, it maps a cluster molecule to another molecule in the cluster. Thus, if a symmetry operator maps at least one cluster molecule to itself, it maps the whole cluster to itself; alternatively, a symmetry operator will map every cluster molecule to a spatially disjoint molecule in the crystal, generating another symmetry-equivalent image of the cluster. There will be therefore 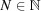 disjoint images of the cluster (without a proof, we note that 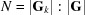). We will further demonstrate that the stoichiometry of the unit cell will only be correct if the stoichiometry of each cluster is correct.

If we use one operator from each left coset of 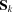 in the cluster symmetry group 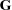 to generate the molecular cluster, then the multiplicity of each general position atom in the cluster is equal to the order of the 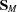 point group of the molecule times the index of the 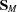 group in the cluster symmetry group, 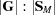, since operators from distinct left cosets of 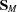 in 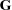 generate distinct molecules. This number is, by Lagrange’s theorem, always the number of symmetry elements in 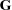. Since the cluster is repeated in the unit cell *N* times, the multiplicity of each general position atom will be 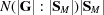. We see that the multiplicity of each atom on a general position in the cluster will be the multiplicity of the atom in the unit cell divided by the same integer *N*. Since the ratios of atoms are stoichiometrically correct in the unit cell, it follows from the above consideration that they were correct in the molecular cluster as well. For atoms on special positions, the same argument applies, with their multiplicities obtained by dividing the general position multiplicity by the special position site symmetry group order. With this, we consider the correctness of stoichiometry in a molecular cluster generated by the above-described rules established.

In both algorithms, atoms are considered as connected by a covalent bond if the distance between them is less than the sum of their covalent radii, plus some extra margin. Atoms are considered overlapping (a ‘bump’) if the distance between them is less than a given fraction of their covalent radii sum. The covalent radii are taken from the work of Pyykkö & Atsumi (2009 ▶) and Cordero *et al.* (2008 ▶), as tabulated by the Blue Obelisk project (O’Boyle *et al.*, 2011 ▶).

## 3. Results   

Both algorithms described above were implemented using the Perl programming language in the *cod-tools* program collection. The molecular symmetry reconstruction and analysis algorithm was implemented as a special mode of the *cif_mol­ecule* program, invoked with the option ‘preserve-stoichiometry’. The expansion to the *P*1 algorithm was implemented both as a second mode of the *cif_molecule* program, invoked using ‘expand-to-P1’, and as a standalone Perl program, *cif_p1*. To test the algorithm, the *cif_molecule* program in both modes was run on all files of revision 117869 of the COD CIF collection. In addition, molecular networks were computed without any use of stoichiometry reconstruction, using a conventional molecular reconstruction algorithm implemented in the same *cif_molecule* program. To test the program, summary chemical formulae were computed for COD entries and compared, the expectation being that both algorithms, if correctly implemented, should yield the same results. The COD in this revision contained 287 301 non-retracted COD CIFs with reported atomic coordinates.^2^ (Here and below, footnotes provide SQL statements used to obtain the quoted numbers from the MySQL database mentioned in this section.) A total of 270 756 summary formulae were computed with all three modes. The results of these computations are available on the COD web site at http://www.crystallography.net/cod/chemistry/formulae/. The difference between the number of COD entries forwarded for processing and the number of computed molecules is caused by the computation time limitation on the computing cluster; most of the CIFs that were not processed contain polymeric molecules with large number of atoms that had to be terminated to make way for the faster and more abundant computations of separate moieties. Since polymeric crystals are not used anyway, the entries that took too much time for computations were discarded at this point of the analysis.

The formulae and polymer flags were loaded into a MySQL database (the database scheme, data load files and Makefiles that produce them from the *cif_molecule* computation results are provided in the supplementary data file^3^), and summary chemical formulae computed by all three algorithms were then compared. The polymeric molecules (a molecule was considered ‘polymeric’ if it had covalent bonds, as detecting by the *cif_molecule* program, in the same molecule related by a crystal lattice translation) were excluded from the analysis, since we did not yet implement an unambiguous way to ‘cut’ polymers into monomeric units. Polymers were excluded by marking them as such during the molecule reconstruction step and storing the mark in a separate database table. After exclusion of polymers,^4^ 222 867 formula entries were left for analysis. From these, 25 COD entries were detected^5^ where the stoichiometric formula did not match the formula computed using the *P*1 expansion. All these cases were examined individually, since they potentially indicate either implementation or more fundamental algorithm design errors. After inspection, all 25 mismatches were found to be due to the peculiarities of the CIFs. One file had cell constants incompatible with the declared crystal symmetry (and with no means to correct the entry). The remaing 24 mismatches were due to crystal disorder: in these CIFs, different disorder groups were reported to have different atomic composition, and thus the formula could not be computed unambiguously. All these 25 entries were commented in a special comments table and excluded from the further analysis.

Finally, the most interesting cases were identified where the stoichiometric summary formula did not match the formula computed by a ‘simple’ algorithm. There were 37 122 such structures under investigation.^6^ These are exactly the cases for which the described algorithm appears to be useful, as in the COD 2231955 example (Fig. 1 ▶).

## 4. Discussion   

The implemented algorithms generate stoichiometrically correct molecular ensembles in a fully automated run from all but 25 nonpolymeric moieties in the COD. The nonpolymeric molecules comprise more than 82% of the COD entries and include such important molecules as drugs, bioactive compounds, organic semiconductors, catalysts and precursors for synthesis. Therefore, automatic extraction of chemical information for this COD subset and linking with chemical databases like PubChem (Bolton *et al.*, 2008 ▶), ChemSpider (Pence & Williams, 2010 ▶) and DrugBank (Law *et al.*, 2014 ▶) and open information sources like Wikipedia is deemed to bring important information about relations between structures and properties of these molecules. The algorithm, unfortunately, will not process correctly many inorganic compounds and metal–organic frameworks, namely the ones that form covalent or coordination polymers in crystals. The proposed algorithm alone, however, cannot solve the problem of monomer identification and must be augmented with heuristics to provide convenient ‘cut’ positions in the covalently connected polymer chains, planes or three-dimensional meshes. The algorithm is run on the COD files to provide cached results for the COD files, but the described programs can be run as standalone processes to process any CIFs.

Despite its inability to process crystal structures of covalent and coordination polymers at present, the algorithm is extremely useful for processing large crystallographic databases like COD. Indeed, we can now generate stoichiometrically correct molecular descriptions (structural formulae, molecular formulae) in bulk for a large subset of published crystal structures and deposit them in chemical databases and/or match them against the chemical database inventories. Such matches will permit large-scale interdisciplinary cross-database searches in various branches of science, benefiting chemists, biologists, pharmacologists and materials scientists, to name representatives of just a few scientific disciplines. The inability to process polymers is not a fundamental limitation of our algorithm: a separate pre-processing step can and will be used to fragment polymer representations into separate monomers. With such an approach, the presented algorithm will provide a stoichiometrically correct representation of a monomer unit. Since the pre-processing does not change the described molecular unit generation method, and since splitting chains into monomers may involve different heuristics and therefore is not unique, we do not include such splitting in the current implementation and do not describe it here in detail.

The algorithm and its implementation are targeted at database maintenance. The strategy here is to process automatically the bulk of the database records and identify the difficult cases so that they can either be processed with other algorithms or, if it is a small number of really difficult cases, be handled by human experts. Automatically processing 222 867 nonpolymer entries (more than 77% out of 287 301 total COD records, with just 25 cases for manual investigation) is for COD maintainers a huge benefit, providing a significant reduction of the human effort. Indeed, finding 37 122 stoichiometrically incorrect structures out of 222 867 nonpolymers by hand would be a prohibitive task for a human maintainer: too slow, too error prone and too costly. With the present algorithm at hand, we can already generate over 222 000 correct structures for, say, deposition to PubChem, coupling our crystallographic information with the wealth of chemical and pharmacological data available there.

When applying the described algorithm to crystal structure analysis, one should take into account a peculiarity of the results when a crystal structure contains more than one molecule (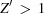) per asymmetric unit (AU), where 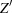 is the number of equivalent molecules in the AU. In such cases, the described algorithm will treat the whole AU as an above-molecular assembly and output it as a formula unit of the crystal. This is because the algorithm assumes that two molecules are the same if they are symmetry equivalent in the crystal, and different otherwise. Such an approach is safe, since we will never declare two different molecules to be identical, by the definition of symmetry equivalence. The algorithm can err, however, to the side of falsely declaring two identical or very similar molecules as different, but this is deemed to be an acceptable compromise since no data are lost in the algorithm’s output and the identical molecules, after choosing the identity criterion, can be filtered away from the output of the molecule reconstruction program. An example of how such filtering can be done is our implementation of the filtering using the Morgan algorithm (Morgan, 1965 ▶). Taking the COD 7151990 entry as an example (Mali *et al.*, 2011 ▶), we obtain the molecular unit formula as 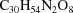 in the default mode (when the Morgan algorithm is not used), corresponding to 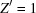, but the molecular unit formula becomes 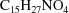 when the Morgan algorithm is employed to detect chemically identical molecules in the output (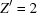). Interestingly, although one could argue that 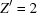 reflects better the real molecular formula of the compound under consideration, the *Z* and summary formula assignment in the supplementary data of the original publication are compatible with 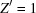. Such examples are common and seem to hint that the default behaviour of the proposed algorithm is the same as in other widely available software packages and is found acceptable by the chemical community.

To summarize the discussion of 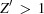 cases, we can postulate that the proposed algorithm maintains a useful invariant: (*a*) its output is a stoichiometrically correct assembly of atoms (molecules), and (*b*) its output contains all covalently connected sets of atoms (‘molecules’ or ‘moieties’) in full, without fragmenting them, and thus is suitable for subsequent processing with chemoinformatics tools. In particular, the output is suitable to derive a formula unit representation and the moiety chemical formula, with an appropriate *Z* according to *International Tables for Crystallography* (Hall *et al.*, 2005 ▶). The ambiguity of the output reflects the ambiguity of *Z* and 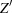 assignment based on crystallographic data alone, but the output contains all necessary information for finding identical molecules (or enantiomers, diastereomers or conformers) according to any set of desired criteria in a post-processing step, thus making the algorithm useful even in cases when more sophisticated molecule identity definitions are necessary.

As expected, the stoichiometric molecule reconstruction process based on symmetry analysis is slightly faster than the algorithm based on expansion to the *P*1 unit cell (Table 1 ▶). The standalone *cif_p1* implementation runs faster than *cif_molecule* when the same expansion to the *P*1 algorithm is used but is still outperformed by the symmetry analysis algorithm in *cif_molecule*. A speed improvement slightly above 25% might not seem significant for single-structure computations; for the whole COD computations, for example for automatic unattended updates of chemical information derived from new database revisions, such speedup is very welcome. Even more importantly, analysis of molecule and crystal symmetry relations gives us a useful tool for representing molecules in a way that is consistent with the needs of both chemical and crystallographic applications.

**Figure 2:**
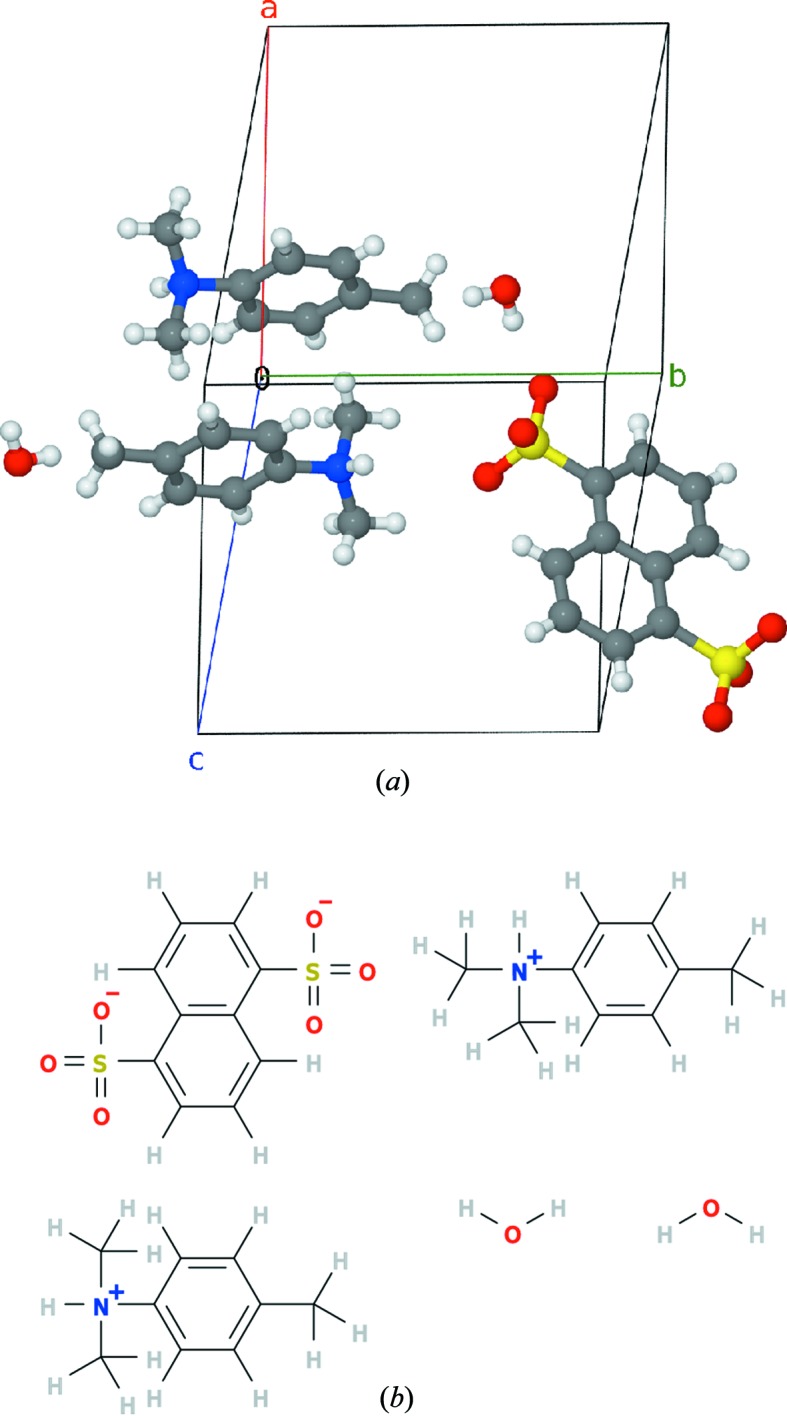
Reconstructions of stoichiometrically correct molecular ensemble from the COD 2231955 entry. (*a*) Molecular cluster reconstructed using the stoichiometry-preserving algorithm. (*b*) The structural formula automatically generated from the reconstructed molecular file. Chemical bond and charge comprehension was performed using the *JUMBO* converters suite (Murray-Rust, 1997 ▶), and then the SVG file was automatically generated using the *Open Babel* package (O’Boyle, Guha *et al.*, 2011 ▶).

Reconstruction of stoichiometric molecules allows automated derivation of correct summary chemical formula and correct moiety formula and depiction of the molecule’s structural formula using automated conversion tools. As seen in Fig. 2 ▶, the algorithm yields correct ratios of anions and cations in an unsupervised run and also correctly indicates that there are two water molecules per naphthalene-1,5-disulfonate moiety in the crystal, which is not immediately apparent from the structure in Fig. 1 ▶.

The algorithm is space-group general and works correctly also in more sophisticated cases. For example, the COD 7115272 structure (Gau *et al.*, 2014 ▶) belongs to the space group 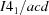 (No. 142), featuring 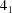, 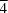 and 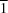 symmetry elements; the metal complex is on a special position with the site symmetry order 4. The described algorithm correctly reconstructs the cluster and the adjacent solvent molecules; the sample implementation code of *cif_molecule* also handles disorder gracefully. The coset algorithm is 1.5 times faster for this structure than the full *P*1 reconstruction (6 *versus* 9 s run time on a laptop with 4 GB RAM and an Intel Pentium CPU B980 running at 2.40 GHz under the Ubuntu 10.04 LTS GNU/Linux 32 bit OS, using software specified in Table 1 ▶).

We thus conclude that the proposed symmetry analysis algorithm can be useful as the first step for automated abstraction of chemical information from the Crystallography Open Database and other crystallographic databases.

## Supplementary Material

Database scheme, data load files and Makefiles. DOI: 10.1107/S1600576714025904/kk5188sup1.zip

## Figures and Tables

**Table:** The cited run times and their standard deviations were computed from five successive runs for 200 randomly selected COD structures on an unloaded Dell Precision T3500 computer with 6GB RAM and the Intel Xeon CPU W3565 running at 3.2GHz, under Ubuntu 12.04 LTS GNU/Linux 32 bit OS, using the distribution’s default Perl interpreter (version 5.14.2), the gcc compiler (version 4.6.3) and *cod-tools* revision 2749.

Program	*cif_molecule* (simple reconstruction)	*cif_molecule* (preserve-stoichiometry)
Run time	4 min 53s 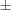 3s	5 min 05s 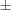 8s

**Table:** 

Program	*cif_molecule* (expand-to-P1)	*cif_p1*
Run time	6 min 55s 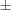 10s	5 min 27s 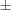 5s

## Footnotes

1 In this manuscript, we use the same definitions as Grosse-Kunstleve (1999 ▶); in particular, a full group is a set of symmetry operations that is closed under binary multiplication, and symmetry operations that are related by combinations of the unit translations are considered to be equal.

2
mysql -u cod_reader -h www.crystallography.net cod -e ‘select count(*) from data where (status not like ‘‘%retracted%’’ or status is null) and (svnrevision <= 117869 or svnrevision is null) and flags like ‘‘%has coordinates%’’

3 Supporting information for this paper is available from the IUCr electronic archives (Reference: KK5188).

4
select count(*) from formulae where is_polymer = ‘‘no’’

5
select count(*) from formulae where is_polymer = ‘‘no’’ and stoichiometric != p1

6
select count(*) from formulae where is_polymer = ‘‘no’’ and stoichiometric != simple and comment is null

## References

[bb1] Allen, F. H., Johnson, O., Shields, G. P., Smith, B. R. & Towler, M. (2004). *J. Appl. Cryst.* **37**, 335–338.

[bb2] Berman, H. M., Kleywegt, G. J., Nakamura, H. & Markley, J. L. (2012). *Structure*, **20**, 391–396 10.1016/j.str.2012.01.010 PMC3501388 22404998

[bb3] Bolton, E. E., Wang, Y., Thiessen, P. A. & Bryant, S. H. (2008). *PubChem: Integrated Platform of Small Molecules and Biological Activities*, Annual Reports in Computational Chemistry, Vol. 4, ch. 12, pp. 217–240. Oxford: Elsevier.

[bb4] Brown, I. D. & McMahon, B. (2002). *Acta Cryst.* B**58**, 317–324. 10.1107/s0108768102003464 12037350

[bb5] Cordero, B., Gómez, V., Platero-Prats, A. E., Revés, M., Echeverría, J., Cremades, E., Barragán, F. & Alvarez, S. (2008). *Dalton Trans.* pp. 2832–2838. 10.1039/b801115j 18478144

[bb6] Dalby, A., Nourse, J. G., Hounshell, W. D., Gushurst, A. K. I., Grier, D. L., Leland, B. A. & Laufer, J. (1992). *J. Chem. Inf. Comput. Sci.* **32**, 244–255.

[bb7] Gau, M. R., Hamilton, C. R. & Zdilla, M. J. (2014). *Chem. Commun.* **50**, 7780–7782. 10.1039/c4cc02872d 24865224

[bb8] Gražulis, S., Chateigner, D., Downs, R. T., Yokochi, A. F. T., Quirós, M., Lutterotti, L., Manakova, E., Butkus, J., Moeck, P. & Le Bail, A. (2009). *J. Appl. Cryst.* **42**, 726–729. 10.1107/S0021889809016690 PMC3253730 22477773

[bb9] Gražulis, S., Daškevič, A., Merkys, A., Chateigner, D., Lutterotti, L., Quirós, M., Serebryanaya, N. R., Moeck, P., Downs, R. T. & Le Bail, A. (2012). *Nucleic Acids Res.* **40**, D420–D427. 10.1093/nar/gkr900 PMC3245043 22070882

[bb10] Grosse-Kunstleve, R. W. (1999). *Acta Cryst.* A**55**, 383–395. 10.1107/s0108767398010186 10927267

[bb11] Hall, S. R., Fitzgerald, P. M. D. & McMahon, B. (2005). *International Tables for Crystallography*, Vol. G, ch. 3.2, pp. 93–107. Heidelberg: Springer.

[bb12] Hanson, R. M. (2010). *J. Appl. Cryst.* **43**, 1250–1260.

[bb13] Hanwell, M., Curtis, D., Lonie, D., Vandermeersch, T., Zurek, E. & Hutchison, G. (2012). *J. Cheminformatics*, **4**, 17. 10.1186/1758-2946-4-17 PMC3542060 22889332

[bb14] Law, V. *et al.* (2014). *Nucleic Acids Res.* **42**, D1091–D1097. 10.1093/nar/gkt1068 PMC3965102 24203711

[bb15] Levinthal, C. (1966). *Sci. Am.* **214**, 42–52. 10.1038/scientificamerican0666-42 5930597

[bb16] Mali, S. M., Bandyopadhyay, A., Jadhav, S. V., Kumar, M. G. & Gopi, H. N. (2011). *Org. Biomol. Chem.* **9**, 6566–6574. 10.1039/c1ob05732d 21826295

[bb17] Morgan, H. L. (1965). *J. Chem. Doc.* **5**, 107–113.

[bb18] Murray-Rust, P. (1997). *World Wide Web J.* **2**, 197–206.

[bb20] O’Boyle, N. M., Banck, M., James, C. A., Morley, C., Vandermeersch, T. & Hutchison, G. R. (2011). *J. Cheminformatics*, **3**, 33. 10.1186/1758-2946-3-33 PMC3198950 21982300

[bb19] O’Boyle, N., Guha, R. *et al.* (2011). *J. Cheminformatics*, **3**, 37.

[bb21] Pence, H. E. & Williams, A. (2010). *Chem. Educ. Today*, **87**, 1123–1124.

[bb22] Persistence of Vision (2004). *Persistence of Vision Raytracer*. Version 3.6. http://www.povray.org.

[bb23] Pyykkö, P. & Atsumi, M. (2009). *Chem. Euro. J.* **15**, 12770–12779. 10.1002/chem.200901472 19856342

[bb24] Wall, L., Christiansen, T. & Orwant, J. (2000). *Programming Perl*, 3rd ed. Sebastopol: O’Reilly Media.

[bb25] Wei, B. (2011). *Acta Cryst.* E**67**, o2678. 10.1107/S1600536811037068 PMC3201563 22065142

